# Visualizing cellular heterogeneity by quantifying the dynamics of MAPK activity in live mammalian cells with synthetic fluorescent biosensors

**DOI:** 10.1016/j.heliyon.2020.e05574

**Published:** 2020-12-07

**Authors:** Min Ma, Pino Bordignon, Gian-Paolo Dotto, Serge Pelet

**Affiliations:** a Department of Fundamental Microbiology, University of Lausanne, Switzerland; b Department of Biochemistry, University of Lausanne, Switzerland

**Keywords:** Cell biology, Systems biology, Biochemistry, Cancer research, MAPK signaling, Single cells, Fluorescent biosensor, Live-cell imaging

## Abstract

Mitogen-Activated Protein Kinases (MAPKs) control a wide array of cellular functions by transducing extracellular information into defined biological responses. In order to understand how these pathways are regulated, dynamic single cell measurements are highly needed. Fluorescence microscopy is well suited to perform these measurements. However, more dynamic and sensitive biosensors that allow the quantification of signaling activity in living mammalian cells are required. We have engineered a synthetic fluorescent substrate for human MAPKs (ERK, JNK and p38) that relocates from the nucleus to the cytoplasm when phosphorylated by the kinases. We demonstrate that this reporter displays an improved response compared to other relocation biosensors. This assay allows to monitor the heterogeneity in the MAPK response in a population of isogenic cells, revealing pulses of ERK activity upon a physiological EGFR stimulation. We show applicability of this approach to the analysis of multiple cancer cell lines and primary cells as well as its application *in vivo* to developing tumors. Using this ERK biosensor, dynamic single cell measurements with high temporal resolution can be obtained. These MAPK reporters can be widely applied to the analysis of molecular mechanisms of MAPK signaling in healthy and diseased state, in cell culture assays or *in vivo*.

## 1 Introduction

Given that cells live in changing environments, they have developed complex biochemical networks to sense and transmit extracellular information. Intensive biological research has allowed the identification of multiple signal transduction pathways that relay the presence of nutrients, stress or hormones in the cell's surroundings. The biochemical analysis of these pathways has allowed the identification of the major players implicated in these processes. However, to fully grasp the complex regulation of these biochemical signals, quantitative and dynamic measurements have to be performed.

An additional layer of complexity arises from the fact that each cell, even in an isogenic population, can display different cellular behaviors [1]. Population-averaged measurements can prevent the identification of important signaling patterns such as oscillations or bimodality [2, 3]. Therefore, the value of single cell measurements has been generally recognized. Microscopy and flow cytometry, as well as novel techniques such as mass cytometry or single cell sequencing, have gained in importance to unravel the biochemical signals taking place in single cells. In the signaling field, the dynamics of the biological system is an essential parameter in the transmission of the biological information and the determination of cellular fate [3, 4]. Live-cell imaging is the only technique that can capture the temporal evolution of individual cells. However, more tools are required to enable the quantification of signaling activity in single cells in order to uncover novel biochemical regulations in signal transduction cascades.

In mammalian cells, quantification of kinase activity is of particular interest for signal transduction studies, because more than one third of cellular proteins have identified phosphorylation sites [5]. Among these kinases, Mitogen-Activated Protein Kinases (MAPKs) play a central role in the signaling network of the cell. These proteins are evolutionary conserved serine/threonine kinases that mediate a wide range of cellular processes, including cellular growth, differentiation, adaptation to stress, apoptosis or motility [6, 7, 8, 9]. MAPKs can be activated by a wide range of extracellular signals. These environmental cues are typically detected by membrane sensors, which relay this information inside the cell to the MAPK signaling cascades. These cascades consist of three-core kinases (MAP3K, MAP2K, and MAPK) with sometimes an additional upstream MAP4K and a downstream MAPK-associate protein kinase. Once activated, the MAPK phosphorylates a wide array of substrates to modulate their activity. A minimal consensus site (SP or TP) is needed for MAPK phosphorylation, therefore the specificity of the process is achieved via distinct protein-protein interaction domains. One conserved interaction motif is the docking site (DS), which is present in many substrates and activators of the MAPKs [10, 11, 12]. In addition to the phosphorylation of substrates, MAPKs are tightly implicated in the induction of specific transcriptional program that can profoundly alter the cellular state.

In mammalian cells, three distinct groups of MAPKs have been described: Extracellular signal-Regulated Kinase (ERK), c-Jun N-terminal Kinase (JNK) and p38 [13, 14, 15]. The analysis of targeted mutations in mice, and the development of specific inhibitors have begun to shed light on the function of these cascades in mammals. As MAPKs regulate multiple central cellular processes, misregulation of MAPK signaling is implicated in the induction and progression of a broad range of diseases. Indeed, deregulation of the ERK pathway has been identified in approximately one third of all human cancers. The ERK pathway has been shown to be implicated in multiple fundamental cellular processes such as cell cycle entry, proliferation and differentiation [16]. In this cascade, ligand-mediated activation of a receptor tyrosine kinase triggers the Ras GTPase, which recruits and activates the MAP3K RAF. In turn, RAF phosphorylates and activates the MAP2K MEK, which finally activates the MAPK ERK. This kinase further relays the signal to many substrates. Among these substrates, there are several key transcription factors that will modulate the expression of genes implicated in cell fate decisions [17, 18].

Due to the important role played by MAPKs in cellular fate determination and cancer progression, the ability to quantify their activity in live single cells is highly required. Numerous FRET bio-reporters have been developed and are commonly used to track the real-time kinase activities in single cells [19, 20, 21, 22]. To obtain a robust FRET signal, a large change in conformation between the active and inactive state of the molecule is required. Unfortunately, in practice these changes are often small and require a tedious optimization procedure to be improved [23]. In addition, the FRET probes block two fluorescent channels of the microscope, therefore limiting their application to monitor multiple cellular activities within the same cell.

Due to the complexity in obtaining reliable FRET measurements, novel biosensors based on fluorescent protein relocation have been developed. One strategy is to modulate the shuttling of a fluorescent protein between the nucleus and the cytoplasm by phosphorylation. In mammalian cells, the KTR (Kinase Translocation Reporter) was developed to monitor ERK, JNK and p38 activities [24]. In yeast cells, the SKARS (Synthetic Kinase Activity Relocation Sensor) has been engineered to monitor MAPK activity in the mating and cell wall integrity pathways [25]. More recently, a new strategy based on protein aggregation has been presented [26], where the phosphorylation of a synthetic substrate by the kinase leads to the formation of bright fluorescent foci. One key advantage of these reporters is that they occupy a single fluorescent channel in the microscope. The combination of multiple fluorescent sensors within a single cell becomes feasible allowing the correlation of various signaling activities within the same cell [24, 27].

In this paper, we have adapted the SKARS approach to quantify ERK, JNK and p38 activities in mammalian cells. We verified that this sensor provides an improved readout compared to the KTR reporter by co-expressing both sensors in the same cells. This biosensor allows to monitor complex signal transduction dynamics in single cells that display a large heterogeneity. In addition, we show that it can be applied to a wide range of cell types *in vitro* and in developing tumors *in vivo*.

## 2 Results

### 2.1 The concept of SKARS biosensors for mammalian MAPK signaling

Nuclear Localization Signals (NLSs) are small amino acid sequences that confer on proteins the ability to be transported in the nucleus. These domains are highly conserved throughout eukaryotes, and for instance the same SV40 NLS motif is commonly used in yeast as well as in mammalian cells. NLSs are positively charged peptides and often consist of a mix of four lysines or arginines. These positive charges enable a strong interaction with the importin, which will promote the transfer of interacting proteins through the nuclear pores into the nucleus [28]. The amount of nuclear enrichment is directly linked to the ability of the NLS to bind to the importin [29]. Point mutations in the NLS consensus sequence introducing uncharged amino-acids decrease the accumulation in the nucleus. Similarly, addition of negatively charged residues in the vicinity of the positively charged patch weakens the nuclear enrichment [30]. Many transcription factors possess phosphorylation sites in the vicinity of an NLS in order to control their nuclear enrichment in response to signaling events [31, 32, 33].

**Figure 1:**
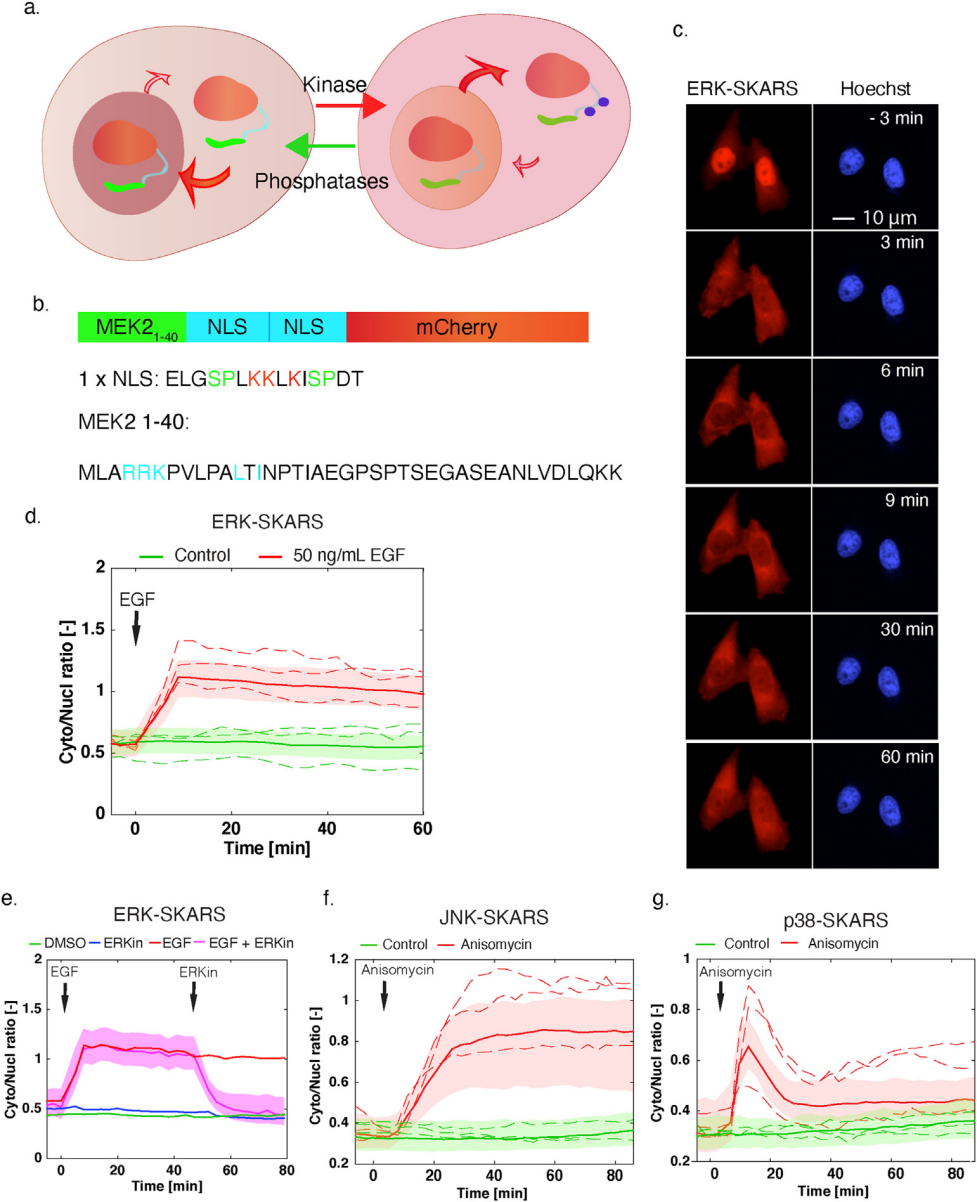
Principle and development of synthetic kinase activity relocation sensor (SKARS) to monitor MAPK activity in mammalian cells. a. Scheme of the SKARS relocation process. When the kinase is inactive, the NLS is functional and the sensor accumulates in the nucleus. When the kinase is active, it phosphorylates the SKARS, which relocates into the cytoplasm. b. The ERK-SKARS contains three domains: the ERK docking site (MEK2 1–40), the two Nuclear Localization Sequences (NLS), and the fluorescent protein for visualization. Residues involved in interaction, nuclear import or phosphorylation are shown in cyan, red and green, respectively. c. Representative microscopy images of HeLa cells expressing the ERK-SKARS cells and stimulated with EGF (50 ng/ml) for 1 h period. In the red channel, the ERK-SKARS translocates from the nucleus to the cytoplasm. Nuclei are identified by a Hoechst staining. d. After quantification of the time-lapse movies, the ratio of the average cytoplasmic fluorescence of the average nuclear fluorescence (Cyto/Nucl ratio) is plotted as function of time. For all similar figures in this paper, the solid lines represent the median of the cell population and the shaded area the 25 and 75 percentiles of the population. The dotted lines represent a few single-cell traces extracted from the cell population. More than hundred single cells measured were plotted in the graph. The red curve represents HeLa cell treated with 50 ng/ml EGF (Number of cells: Nc = 340) and the green curve, mock-treated control cells (Nc = 449). e. Cyto/Nucl ratios of HeLa cells expressing the ERK-SKARS exposed to EGF stimulation (50 ng/ml) and ERK inhibition (FR 180204, 50 ng/ml) are plotted as function of time. EGF and ERK inhibitor were added at the time points indicated by the arrows. f. and g. HeLa cells expressing the JNK-SKARS (f) and the p38-SKARS (g) were treated with (red) and without (green) Anisomycin (50 ng/ml). The Cyto/Nucl ratio is plotted as function of time.

Our SKARS biosensors aim at mimicking this behavior. We express in the cell a synthetic substrate for a MAPK that can shuttle between the cytoplasm and the nucleus based on its NLS phosphorylation status. We combine two NLSs in order to promote a strong enrichment of the biosensor in the nucleus under basal conditions. Both NLSs bear two MAPK consensus phosphorylation sites (SP). The specific phosphorylation of the serines is controlled by the presence of a MAPK docking site (DS) placed nearby [34, 35]. These short peptides forming the docking modules are well known to steer the enzymatic activity of the MAPK towards specific proteins and exchanging these sequences between different proteins will alter the regulation by the MAPK [10]. Finally, a fluorescent protein is functionalized with the DS and the NLSs to generate our reporter. In the absence of kinase activity, the fluorescence signal will accumulate in the nucleus thanks to the NLSs; while in the presence of an active MAPK, the phosphorylation of this synthetic substrate will result in its relocation into the cytoplasm (Figure 1a).

### 2.2 Quantification of endogenous ERK activity by the ERK-SKARS

In order to adapt our previously developed yeast SKARS biosensor to report on mammalian MAPK activity, only the MAPK specific interaction module had to be changed. The 2xNLS peptide and the fluorescent proteins remained identical. Remarkably, the ability to use the same functional part of the sensor strongly suggests that this reporter could be applied to a diverse range of eukaryotes. The docking site, however, should be adapted for each MAPK of interest to ensure the specificity of the interaction. In mammalian cells, these interaction domains have been extensively studied and many candidate DS motifs are described in the literature. In order to target the ERK pathway, we selected a DS for ERK2 on its direct upstream activator MEK2, using the first 40 amino acids of this protein (Figure 1b) [35].

The ERK-SKARS was cloned into a lentiviral vector and lentiviruses were produced to infect HeLa cells to generate cells stably expressing our construct. As shown in Figure 1c, the inactive form of the sensor accumulates in the nucleus of HeLa cells incubated in starved medium. Upon EGF stimulation (50 ng/ml), the ERK signaling cascade is activated and the MAPK can phosphorylate the serines in the vicinity of the NLS on the sensor. This leads to a rapid redistribution of ERK-SKARS to the cytoplasm. As illustrated in Figure 1c, six minutes after the stimulus, the nuclei of the two cells from the picture are strongly depleted from the bioreporter, providing a qualitative readout of MAPK activity in these cells.

In order to obtain quantitative measurements, time-lapse movies were automatically analyzed [36]. A DNA staining dye (Hoechst) was used to automatically segment the nuclei, allowing to quantify the fluorescence intensity of the SKARS in the nucleus. The intensity in the cytoplasm is extracted from a ring surrounding the nucleus. The ratio of the cytoplasmic over nuclear intensities in each single cell is used as a proxy for MAPK activity. In Figure 1d, the median and 25-, 75-percentiles of the population response are plotted for more than 300 single cells stimulated with 50 ng/ml EGF and for an unstimulated control (PBS). Before stimulation, the ERK-SKARS is enriched in the cell nucleus, therefore the cytoplasm to nucleus ratio (Cyto/Nucl) is low (below 1). After EGF addition, the ratio increases suggesting that the sensor has been phosphorylated by active ERK in response to the EGF stimulation, leading to its shuttling from the nucleus to the cytoplasm. The ratio remains high for the 60-minute duration of the time lapse (Figure 1 c and d). This sustained activation of ERK signaling upon high EGF treatment is also observed by Western blot and immunofluorescence staining (Sup Figure 1). These two techniques are routinely used to quantify ERK activity but they provide snapshot measurements of the cellular signaling state at specific time points. In comparison, the SKARS reporter provides a dynamic readout of kinase activity in live single cells.

### 2.3 Specificity of the ERK-SKARS

To demonstrate the specificity of our SKARS readout, we performed a number of control experiments. First, the four serines present in the NLSs were mutated to either non-phosphorylable (NLS-4A) or phospho-mimicking mutants (NLS-4E) (Sup Figure 2a and b). As expected, the NLS-4A is constitutively enriched in the nucleus (low Cyto/Nucl ratio), while the NLS-4E is depleted from the nucleus (high Cyto/Nucl ratio). In addition, both mutants lose the ability to respond to the EGF stimulus, thereby confirming that the phosphorylation of the four serines is controlling the nuclear localization of the sensor. Second, the five key residues in the docking site were mutated to alanine (non-docking MEK2, MEK2_ND_) (Sup Figure 2c). In the same cells, we compared the response of the non-docking mCherry SKARS and a functional sensor with a GFP fluorophore. While the reporter bearing the DS of MEK2 displayed the expected relocation from the nucleus into the cytoplasm, the ND reporter localization remained unchanged upon the addition of EGF (Sup Figure 2d to f), indicating a clear specificity of the substrate for the kinase interaction and phosphorylation of the substrate. Interestingly, these experiments also demonstrate that, while mutations in the DS or the NLS alter the function of the sensor, the fluorescent protein can be exchanged without perturbing the ability of the reporter to relocate. This feature allows to rapidly adapt the SKARS to specific imaging requirements and enables the combination of multiple sensors in the same cell.

To further validate the specificity of the ERK-SKARS reporter, a direct selective inhibitor of ERK (FR180204 [37]) was applied to block the phosphorylation activity of the MAPK. The addition of the ERK inhibitor 45 min after the EGF stimulus led to a rapid return of the sensor into the nucleus (Figure 1e). In comparison, ERK activity was sustained for more than 80 min in the control cells. In addition, pretreatment of the cells with the same ERK inhibitor abolished the EGF-stimulated translocation of the ERK-SKARS (Sup Figure 3a). In contrast, cells pretreated with JNK or p38 inhibitors displayed an unperturbed relocation of the ERK sensor upon EGF stimulation (Sup Figure 3b and c). Taken together, these results demonstrate that the ERK-SKARS can efficiently and reliably quantify ERK kinase activity in live single mammalian cells.

### 2.4 JNK and p38 SKARS

In order to change the specificity of a substrate from ERK towards JNK or p38, its docking site can be swapped [35, 38]. We selected docking sites present in downstream transcription factors of the two MAPKs (c-Mef2 for p38 and c-jun for JNK) to replace the MEK2 docking sequence of the ERK-SKARS (Sup Figure 4). Using lentiviral vectors, we generated HeLa cell lines stably expressing either JNK-SKARS or p38-SKARS. As expected, anisomycin stimulation resulted in a fast translocation of both sensors into the cytoplasm (Figure 1f, g, and Sup Figure 4). Interestingly, the dynamics of the responses were very different for the two kinases. While anisomycin induced a sustained activation of the JNK pathway, p38 signaling was only transiently activated for 30 min, in agreement with previous observations [39]. This experiment demonstrates the robustness and versatility of these relocation sensors. Modulating their specificity simply by the substitution of the docking site, offers the opportunity to target kinases with similar modes of action (for example CDK) or adapting the reporter to other organisms.

### 2.5 Comparison of KTR and SKARS for the quantification of kinase activity

**Figure 2:**
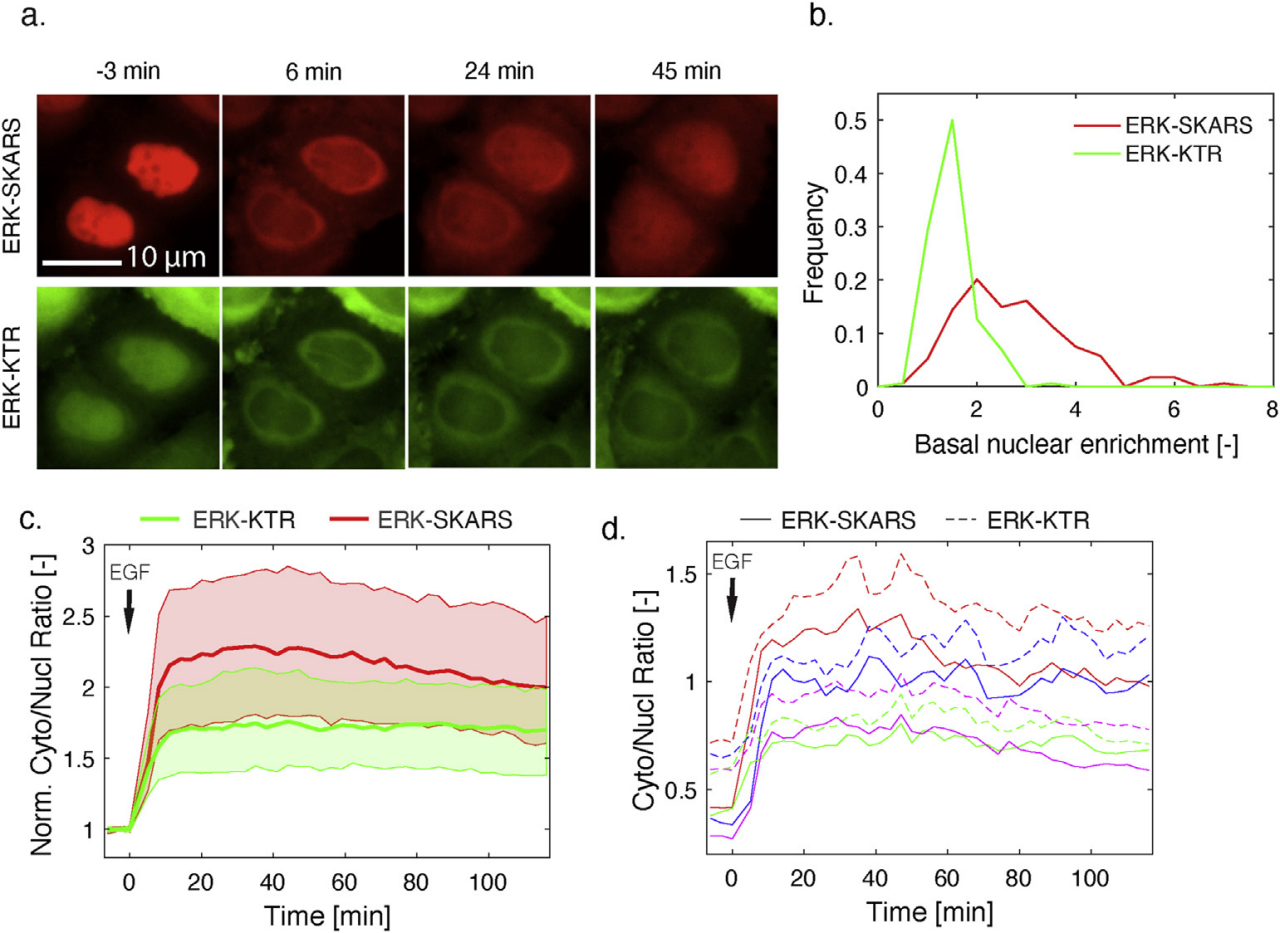
Direct comparison of ERK-SKARS and ERK-KTR in the same cells. a. Representative microscopy images of single HeLa cells carrying ERK-SKARS (red) and ERK-KTR (green) exposed to stimulation (EGF 50 ng/ml) and imaged at indicated time points. b. Histogram of SKARS and KTR nuclear enrichments before (Nucl/Cyto) of HeLa cells expressing ERK-SKARS (red) and ERK-KTR (green) before stimulation (mean of three first time points). c. Median (solid line) and 25- to 75-percentile (area) of the Cyto/Nucl ratios from ERK-SKARS (red) and ERK-KTR (green) upon stimulation by 10 ng/ml EGF. The single cell traces were normalized to the basal level (the mean of the first 3 three time points) (Nc = 234). d. Single cell traces of the Cyto/Nucl ratios for ERK-SKARS (solid line) and ERK-KTR (dashed line) were plotted as function of time. Each color represents the measurements from one single cell, and both reporter traces for each cell are displayed.

The design of our sensors bears many similarities with the KTR system [24]. Therefore, in order to compare the relative abilities of the SKARS and the KTR to quantify MAPK activity, we generated cells stably expressing both reporters (Figure 2a). Both sensors were made spectrally compatible with an ERK-SKARS based on an mCherry protein, while the ERK-KTR was coupled to an mClover protein (a GFP variant). These two constructs are based on two different ERK DS: MEK2 for the SKARS and ELK1 for the KTR. But the major difference between these reporters resides in the peptides that control the nuclear to cytoplasmic shuttling. Indeed, the KTR combines an NLS and an NES (Nuclear Export Signal). The kinase activity drives the export of the construct because the phosphorylation weakens the NLS and enhances the efficiency of the NES. In comparison, for the SKARS, only the nuclear import rate is modulated by the phosphorylation of the NLS, and the sensor relies on the natural diffusion through the nuclear pores to exit the nucleus.

To standardize the expression levels of both reporters, we focused our analysis on cells expressing both constructs at intermediate levels (Sup Figure 5a). In these cells, the nuclear enrichment of the SKARS in basal condition is higher than for the KTR (Figure 2b, Sup Figure 5b, Sup Table 1). In order to directly compare the responses of both sensors in the same cell, single cell traces were normalized to their basal levels (mean of the first three time points before stimulus). As shown in Figure 2c, when cells were stimulated with EGF, both ERK-SKARS and ERK-KTR sensors could rapidly translocate from the nucleus to the cytoplasm, leading to a rapid increase of cytoplasm to nucleus ratio (Figure 2c and Sup Figure 5c). However, relative to the ERK-KTR population response amplitude (1.5 folds on average), the cytoplasm-to-nucleus ratio change is larger for the SKARS sensor (2 fold on average). At the single-cell level, the traces for both sensors in the same cell were comparable but the amplitude of the SKARS response was generally larger (Figure 2d and Sup Figure 5d). Similar results were obtained when comparing the JNK-SKARS and the JNK-KTR in the same cells (Sup Figure 6). Note that in the case of the JNK reporters, both sensors are based on the same docking site, thus the difference in relocation observed stems only from the phosphorylated part of the sensor. Because both techniques measure the ability of the MAPK to promote the translocation of the reporter out of the nucleus, starting with a higher nuclear pool of SKARS sensors provides a better dynamic range for the SKARS design compared to the KTR system.

### 2.6 Single cell analysis of the dynamics of ERK activity

After validation of the ERK-SKARS reporter in mammalian cells, we wanted to assess how much heterogeneity in ERK activity existed within a population of isogenic cells. In order to achieve this, we isolated single-cell clones by serial dilution (See Methods). Three different cell lines bearing the ERK-SKARS were isolated and imaged. Since they displayed similar behaviors (data not shown), the data from only one of these lines are presented here.

**Figure 3:**
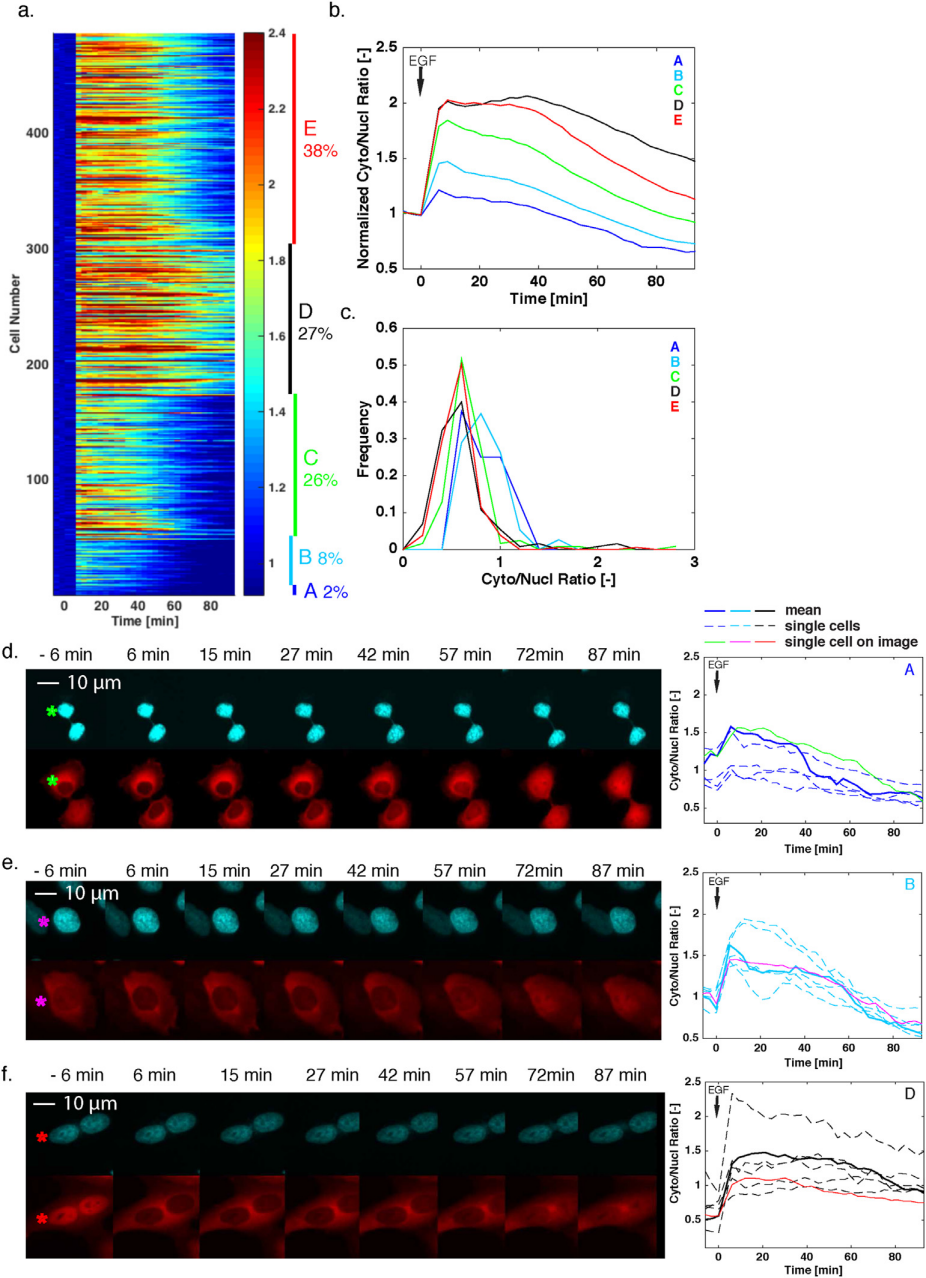
Cellular heterogeneity in ERK activity upon stimulation with high concentration of EGF. a. Heat map of the response of individual cells to a stimulation with EGF (50 ng/ml) in single clonal HeLa cells expressing the ERK-SKARS. Each line represents the cytoplasmic to nuclear ratio of one single cell normalized to the first three time points before stimulation. The cells were sorted using k-mean clustering (See Methods). The different sub-populations are identified by the five colored bars on the right of the map. b. Median of the normalized Cyto/Nucl ratios from the indicated subgroups identified in the population. c. Histograms of the basal Cyto/Nucl ratios measured in the five subgroups. d., e. and f. Representative microscopy images of HeLa cells expressing the ERK-SKARS from three different sub-populations (A, B, D). The right panel displays the Cyto/Nucl measurements of five single cells from that sub-population (dashed lines). The thin solid line corresponds to the single cell identified in the microscopy images on the left with an asterisk. The thick solid line represents the mean response from the sub-population.

First of all, we noticed that individual cells displayed a large variability of nuclear sensor enrichment before the EGF stimulation in starved medium, suggesting that different basal activities of ERK are present in each cell. Despite this diversity, upon stimulation with a saturating concentration of EGF, the majority of the population displayed a strong ERK activation. In order to detect different activity patterns in the cellular responses, the cytoplasm to nucleus ratios of the cells were normalized between their basal values (average of the first three time points) and maximum values. K-mean clustering was applied to separate the traces into four sub-populations. A fifth sub-population consists of non-responding cells (relocation less than 0.05) has been removed from the analysis prior to the k-mean clustering. Figure 3a displays the response of almost 500 cells quantified in this experiment in a heat map. Each line corresponds to the normalized cytoplasm to nucleus ratio of a single cell. The traces were grouped in their individual clusters. Figure 3b displays the median dynamics of ERK activation in the five sub-populations.

Interestingly, the two sub-populations with the weakest responses (Figure 3d, e, clusters A and B) display a higher basal cytoplasm to nucleus ratio (Figure 3c). This suggests that these cells already possess a substantial ERK basal activity and further activation of the pathway with EGF only leads to a minor increase in kinase activity. In the other three clusters (Clusters C, D and E, Figure 3f and Sup Figure 7a and b), a strong activation of the pathway is observed with some variability in the dynamics of signaling activity following the stimulus for each cluster. Heterogeneity is present within a population of isogenic cells in part due to differences in cell-cycle stages [40] and the SKARS offers the ability to visualize cell-to-cell variability and understand how it can impact the cellular response.

### 2.7 Low doses of EGF induce a pulsatile response of the ERK pathway

**Figure 4:**
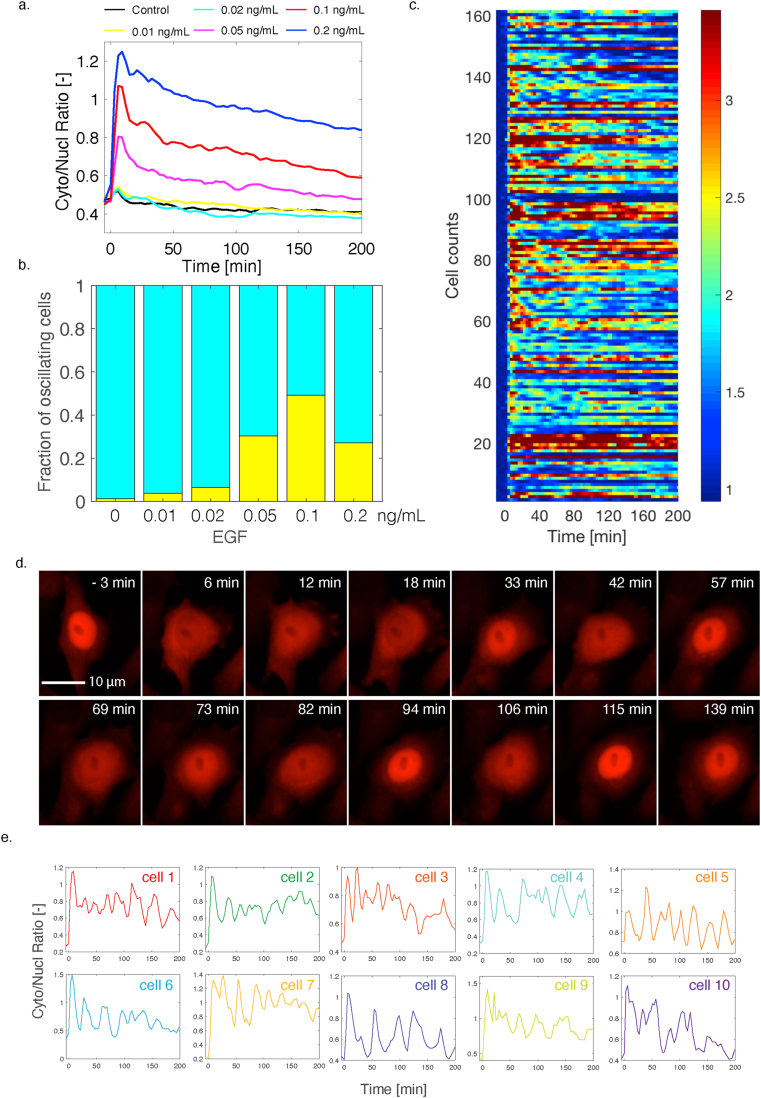
Monitoring of ERK activity pulses upon low doses of EGF. a. Median Cyto/Nucl ratio of ERK-SKARS in more than 400 single cells from a clonal HeLa cell population stimulated with low doses of EGF and monitored for more than 3 h b. Fraction of cells identified in the experiment from panel 4a that display three pulses or more in ERK activity. c. Heat map of the Cyto/Nucl ratio measured upon 0.1 ng/ml EGF treatment in HeLa cells. Only cells where three or more pulses were identified are displayed in this graph. d. Microscopy images of HeLa cell displaying pulses in ERK activation upon 0.1 ng/ml EGF stimulation. e. Various examples of single cell traces displaying fluctuations in ERK activation upon 0.1 ng/ml EGF treatment.

We next wanted to assess the activity of the ERK pathway at more physiological concentrations of EGF [41, 42]. The clonal cell line expressing the ERK-SKARS was stimulated with EGF concentrations ranging from 0.01 ng/ml to 2.5 ng/ml (Figure 4a and Sup Figure 8a–c). Interestingly, around 0.1 ng/ml EGF, in a sub-population of the cells, strong oscillations in ERK activity are observed. At 0.1 ng/ml EGF, almost half of the population display at least three peaks (Figure 4b, Methods). At lower or higher concentrations, the oscillatory behavior tends to disappear and no oscillations were detected in cells stimulated with EGF 2.5 ng/ml or higher. The heat map in Figure 4c represents the cytoplasmic to nuclear ratio of the sub-population of cells stimulated with 0.1 ng/ml where three or more peaks were identified. While the oscillations of individual cells can be visualized in this heat map, no global pattern emerges, indicating an asynchronous oscillation of the cells in the population. Indeed, when plotting individual single cell traces or looking at individual cells (Figure 4d,e, + Sup Movie 1), the ERK activity pulses seem highly stochastic.

The following are the supplementary data related to this article:

SupplementaryMovie_1.mov SupplementaryMovie_1.mov

Multiple studies have uncovered oscillations in ERK activities [43, 44, 45, 46]. Some of these oscillatory patterns were observed with periods on the hour time-scale and were linked to the proliferation of the cells. The behavior we observe is happening on the tens of minutes time-scale. It is reminiscent of the ERK nuclear relocation pulses observed by Shankaran and co-workers when monitoring the MAPK tagged with YFP upon stimulation with 1 ng/ml EGF [46]. Interestingly, this signaling pattern is a unique feature of the EGF response. Cells stimulated with FGF or PDGF did not display this oscillatory behavior (data not shown). This fast translocation of the SKARS between the nucleus and the cytoplasm allows to monitor in real time the fluctuations in MAPK activity.

### 2.8 SKARS in various cells types

**Figure 5:**
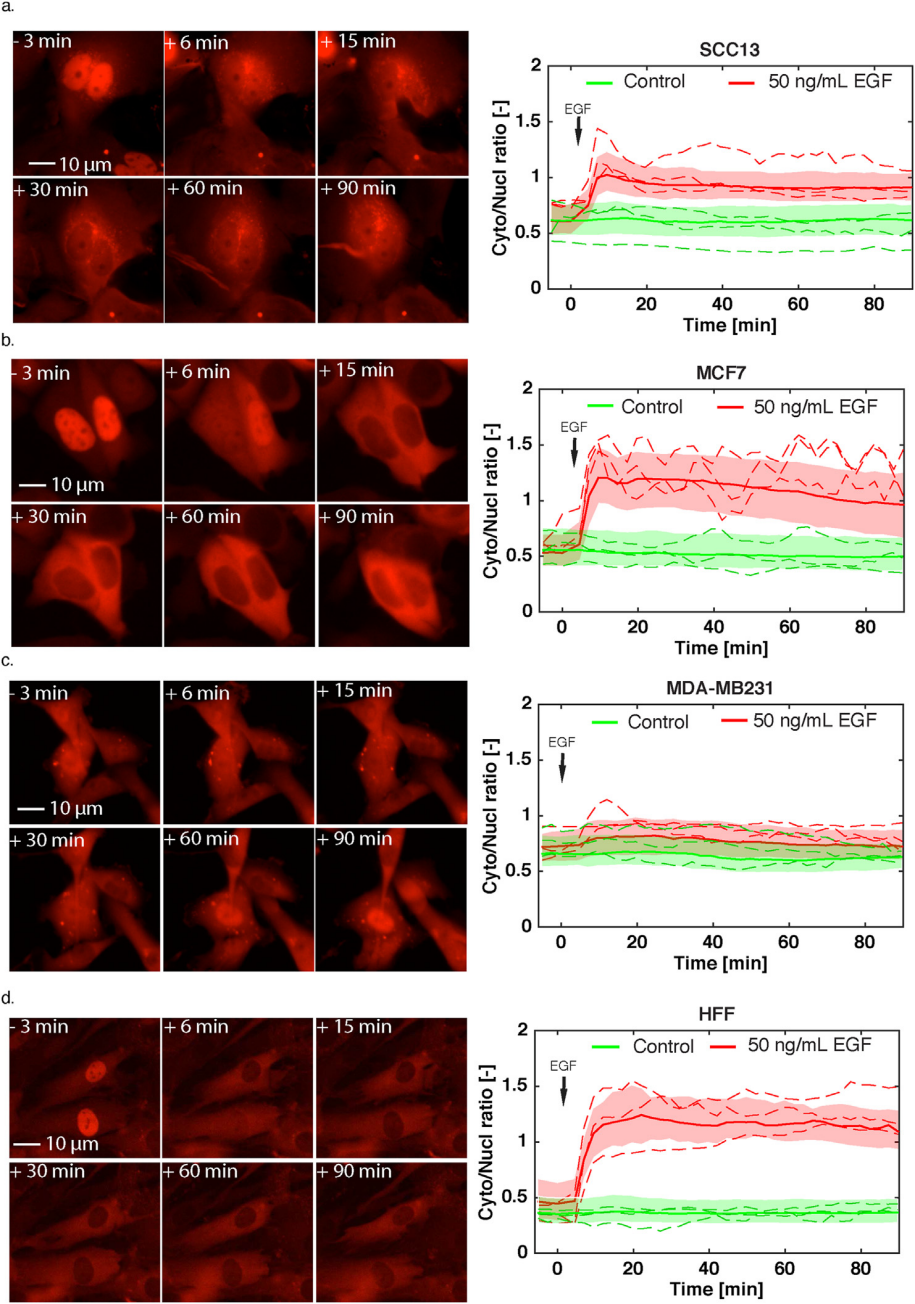
SKARS measurements performed in cancer and primary cell lines. a to d. Left panels show representative microscopy images of SCC13 (a), MCF7 (b), MDA-MB231 (c), HFF (Human Foreskin Fibroblast) (d) cells expressing the ERK-SKARS and stimulated with EGF (50 ng/ml). The right panels show the Cyto/Nucl ratio in the respective cell lines. Quantifications of the time-lapse movies for samples treated with EGF are plotted in red and control samples in green. For each trace, more than 100 single cells measurements were combined to generate the graphs.

All the above results show that the SKARS biosensors provide an efficient and reliable strategy to quantify the MAPK activity. In order to demonstrate the applicability of our sensor to various cell types, we tested the ERK-SKARS in cancer cell lines (Figure 5a–c), primary cells (Figure 5d), primary neuronal cells, as well as, stem cells (data not shown). In the MDA-MB231 cancer cell line, we do not observe a response to EGF stimulus of the ERK-SKARS reporter because this cell line lacks HER2 (Human Epidermal growth factor Receptor 2) expression [47]. Thus, EGF stimulation cannot induce ERK signaling activation (Figure 5c). In all other cell types tested, a strong and sustained response to EGF stimulus is observed and large heterogeneity in the single cell responses can be monitored, as previously observed in the HeLa cells. These results confirm that the SKARS reporter offers a robust assay to quantify ERK activity in diverse samples.

### 2.9 In vivo measurement of kinase activity with ERK-SKARS

Misregulation of MAPK signaling has been observed in numerous diseases, especially in human cancers [48, 49]. A better understanding of cancer progression as well as MAPK pathway target therapy will undoubtedly require the ability to study signaling pathway activity in developing tumors. Currently, the most common way to quantify kinase activity in tissues remains immunohistochemistry. It is a complex and time-consuming method whose sensitivity is highly dependent on the specificity of the antibody and on sample preparation. Moreover, it only provides a snapshot measurement of signaling activity in the tissue. With the advent of novel microscopy techniques, it becomes feasible to image living cells directly in these complex environments [50, 51, 52].

**Figure 6:**
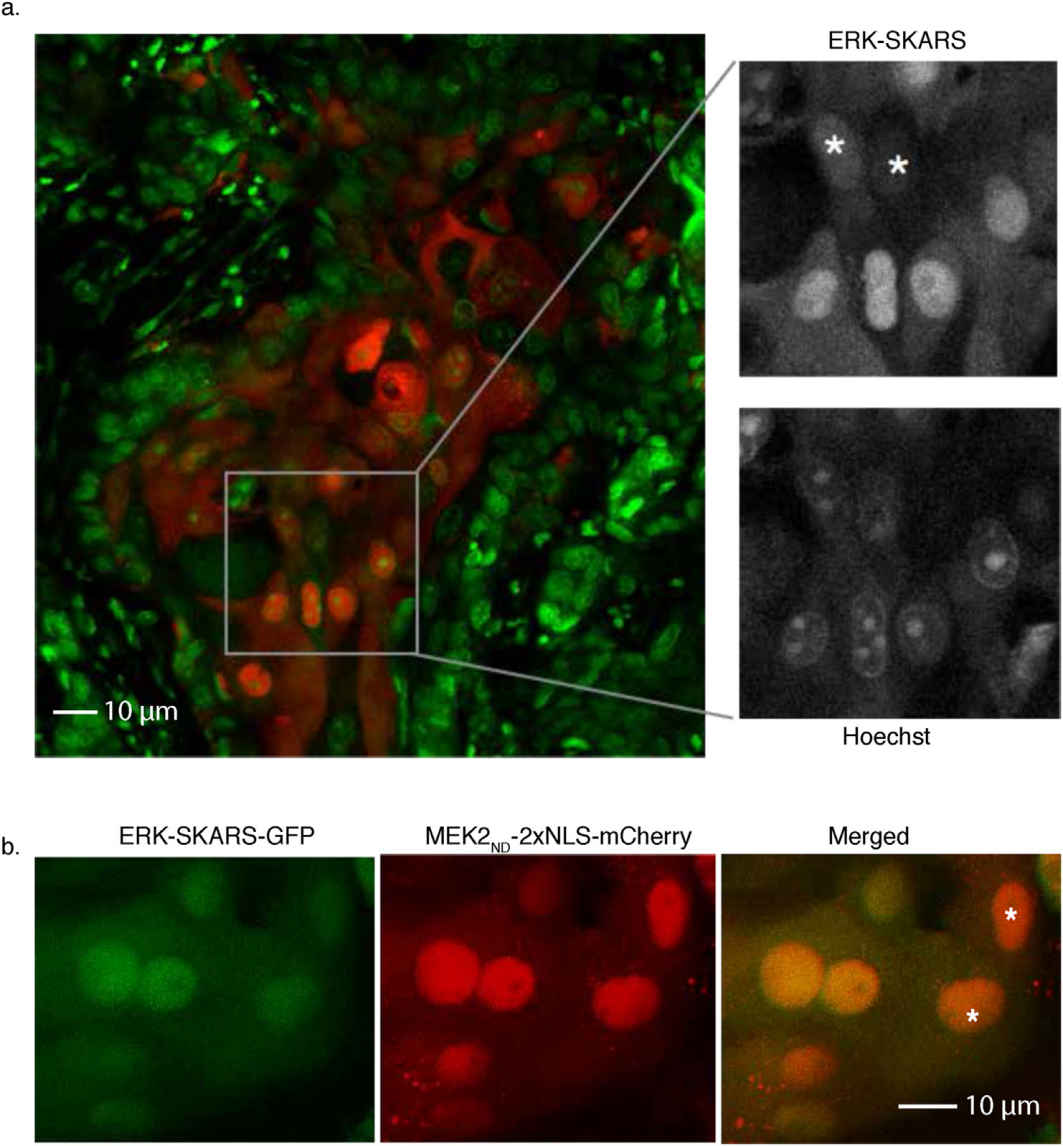
Application of SKARS for kinase activity quantification in tissue. a. Confocal images of HeLa cells expressing ERK-SKARS (red) in tumor implanted in a mouse ear. The green channel represents the nuclei of the cancerous and normal tissues, which were stained with Hoechst. The inset is a magnification of the image to display cells with different levels of nuclear accumulation of relative to the nuclear staining. Asterisk highlight cells with higher ERK activity. b. Confocal images of SCC13 cells expressing ERK-SKARS-GFP (MEK2_DS_, green) and MEK2_ND_-2xNLS-mCherry (red) in tumor implanted inside of mouse ears. Asterisk highlight cells with higher ERK activity.

To test the application of the ERK-SKARS *in vivo*, we used a skin cancer orthotropic model, based on mouse ear intradermal injections of tumorigenic Squamous Cell Carcinoma (SCC) cells [53]. We injected SCC13 cells expressing ERK-SKARS into the ears of 8- to 10-week-old female mice (Severe Combined Immuno Deficiency, SCID, CB17sc-m). After 14 days of growth, the cells formed a small tumor. The tissue was excised into 10–20 μm slides by Vibratome (Leica VT1200/S), stained with Hoechst and then imaged by confocal microscopy. Figure 6a shows the SCC13 cells (red) surrounded by the mouse-ear tissue (green). SCC13 cells displayed different levels of nuclear enrichment of the sensor *in vivo*, indicating that ERK is activated to different extents in these cells. In order to verify the activity of ERK in these settings, we performed another experiment where the SCC13 cells injected in the mouse ear were expressing a functional (MEK2_DS_) and a non-functional (MEK2_ND_) reporter (Figure 6b) as an internal control. In this situation, the lower nuclear enrichment of the ERK-SKARS in the GFP channel relative to the control in the RFP channel can provide a direct measure of kinase activity *in vivo*. The robustness of the SKARS reporter and the simplicity of the activity read-out will allow to expand the ability to monitor MAPK signaling in cancer progression or during development.

## 3 Conclusion

The SKARS and KTR relocation strategy provides a global measurement of kinase activity in a cell in real time. With the addition of a nuclear marker, the quantification of signaling dynamics in hundreds of single cells can be automated. In comparison, the SPARK reporter displays a clear visual readout for MAPK activity. However, with this assay, the quantification of the signal is more challenging due to a non-uniform spatial readout, which complicates its automated measurement in single cells. FRET biosensors can also provide quantitative readout of signaling activity. Compared to the sensors cited above, it has the unique ability to monitor local changes in kinase activity [54, 55]. For some studies, FRET biosensors might therefore have the advantage of adding a spatial information for signaling activity. However, in more complex imaging conditions, such as tissues or in changing environment, the SKARS can potentially offer a better option by providing a robust readout of MAPK activity. In addition, multiple relocation biosensors can be combined in the same cell, allowing to monitor multiple steps within a signal transduction cascade or to correlate the signaling dynamics between different pathways.

## 4 Methods

Supplementary Table 2 summaries the chemical reagents and biological materials used in this study.

### 4.1 Mouse strain

NOD/SCID mice with interleukin-2 receptor gamma chain null mutation (Il2rg −/−) were maintained in the animal facility of the University Lausanne. All mouse work was carried out according to the Swiss guidelines and regulations for the care and use of laboratory animals, with approved protocol from the Canton de Vaud veterinary office.

### 4.2 Human samples

Primary Human Foreskin Fibroblasts (HFF) were extracted from the discarded human tissue samples obtained from the Department of Pediatrics, at Lausanne University Hospital, with institutional approval and informed consent as part of institutional requirements.

### 4.3 Cell culture

The cell lines and the primary cells were cultured and passaged in Dulbecco's Modified Eagle's Medium (DMEM) supplemented with 100 μg/ml streptomycin, 100 units/ml penicillin, 0.25 μg/ml amphotericin B (15240062, Gibco) and 10% heat inactivated Fetal Bovine Serum (FBS, 10270106, Gibco).

### 4.4 Plasmids construction

All the SKARS plasmids for this study are constructed following to standard molecular biology protocol and the vector sequences were confirmed by exhaustively sequencing the cloned fragment. SKARS plasmids were constructed by cloning the docking site for the kinase of interest, the synthetic 2xNLS and the fluorescent protein of the sensor into a Lentiviral Vector backbone plasmid (pLV, a generous gift from Olivier Pertz, University of Bern).

### 4.5 Cell line generation

Stable cell lines expressing the sensor of interest were generated by lentiviral transduction. Lentiviral vectors together with third generation packaging plasmids were transfected into 293T cells to generate lentiviruses. Packaged lentiviruses containing supernatants were added to recipient HeLa cells in the presence of 10 μg/ml Polybrene (AL-118, Sigma-Aldrich). At 3–5 days post transduction, cells were sorted based on fluorescent protein expression by flow cytometry (FACSAria III, BD Biosciences). After sorting, cells were cultured in DMEM. This medium is referred to as complete medium. The starvation medium is completed only with 0.5% FBS. Cells were cultured at 37 °C in a humidified atmosphere containing 5% CO_2_. Singe-cell clones were isolated by serial dilutions of cells in a 96-well plate. To make sure that the colonies arose from single cells, the single-cell colonies isolated have been re-cloned for a second time.

### 4.6 Sample preparation and time-lapse imaging

The day before the time-lapse experiment, 10,000 cells/well of HeLa cells expressing the desired constructs were seeded onto the 96 well glass bottom optical imaging microplate (MGB096-1-2LG, Matrical Bioscience) coated with 10 μg/ml fibronectin (33010-018, ThermoFisher). 2 hours before the microscopy experiment, medium was replaced with Gibco FluoroBrite DMEM medium (A1896701, Gibco) supplemented with GlutaMAX, HEPES, sodium pyruvate and 0.5% FBS. For nucleus staining, we incubate the cells with 10 ng/ml Hoechst 33342 (H3579, Molecular Probes) for 1 h.

Images were acquired on a fully automated inverted epi-fluorescence microscope (Ti-Eclipse, Nikon) controlled by micro-manager [56] using a 20X air objective and appropriate excitation and emission filters. The excitation is provided by a solid-state light source (SpectraX, Lumencor). The images were recorded with an sCMOS camera (Flash 4.0, Hamamatsu). A motorized XY-stage allowed recording multiple fields of view at every time point. GFP (50 ms), RFP (50 ms), DAPI (30 ms) and a bright-field (30 ms) images were recorded at time intervals varying from 2 to 3 min. During the experiments, the temperature (37 °C), the CO_2_ (5%) and the humidity (95%) were kept constant using a temperature-controlled chamber and a CO_2_ controller (Cube and Brick, Life Imaging Service). Stimulations and chemical inhibitors were carefully added to the cells in the incubation chamber after the time-lapse imaging started. To activate ERK signaling, 50 ng/ml (or lower concentrations, where specified) EGF was added. To demonstrate the specificity of the SKARS-ERK, cells were pre-incubated with DMSO, MEK inhibitor (PD032591 100 nM), p38 inhibitor (p38 inhibitor 10 μM) and JNK inhibitor (JNK inhibitor VIII, 10 μM) for 30 min before imaging and stimulation. To inactivate the ERK signaling, ERK inhibitors were added 45 min after the EGF stimulation. To activate JNK and p38 signaling, Anisomycin 50 ng/ml was added to the cells.

### 4.7 Data analysis

Time-lapse movies were analyzed with the YeastQuant platform [36]. The cell nucleus and a 10-pixel-wide cytoplasm ring were segmented for each cell using the Hoechst image as a reference and quantified in each channel. The cell nucleus was tracked across all the frames of the movie. Multiple features of each cell were quantified. For each cell, the average nuclear intensity and the 10-pixel-wide cytoplasm ring in the fluorescent channel corresponding to the SKARS signal were extracted and used to calculate the cytoplasm to nucleus ratio providing a measure of kinase activity in each cell.

Data were further processed using Matlab software (R2017b, MathWorks). For quality control, among all cells tracked over the whole time-lapse experiment, only the cell traces with low variability in nuclear size and intensity were kept for further analysis. The basal level was calculated as the mean of the first three time points of the cytoplasm to nucleus ratio. To cluster the cellular responses in different sub-populations, the non-responding cells were first identified by selecting cells with a change in Cyto/Nucl ratio smaller than 0.05. The other single cell traces were normalized between basal and maximal Cyto/Nucl ratio after stimulus. These normalized traces were fed to the *kmeans* function and the best clustering from 50 iterations was selected. In order to identify the pulses in the traces at low EGF concentrations, the single cell traces were passed to the *findpeaks* function. Only pulses (=peaks) with a minimal prominence of 0.2 were taken into consideration.

### 4.8 Immuno-blotting (IB)

Total proteins were extracted with modified RIPA lysis buffer (50 mM Tris pH 7.4, 150 mM NaCl, 1 mM EDTA pH 8.0, 1% 1X NP40, 0.25% NA-deoxycholate, 2 mM Na-vanadate, 5 mM NaF, 1X protease inhibitors cocktail B (Santa Cruz, sc-45045)). Proteins were separated by SDS-PAGE gel, transferred onto PVDF membrane (Millipore), probed with primary antibody followed by HRP-linked secondary antibodies and detected by SuperSignal west pico chemiluminescent substrate (Thermo Scientific). The primary antibodies used for immunoblotting were rabbit anti-ERK1 Antibody (C-16) Santa Cruz sc-93 (1:1000), ERK2 Antibody (C-14) Santa Cruz sc-154 (1:1000), anti-GAPDH Antibody FL-335 Santa Cruz sc-25778 (1:1000), and anti-Phospho-p44/p42 MAPK (Erk1/2) Antibody (Thr202/tyr204) (D13.14.4E) XP Rabbit mAB (Cell Signaling, 4370). Anti-rabbit antibody (Rabbit IgG, HRP-linked whole Ab from donkey, Amersham NA 934) was used as a secondary antibody. After the detection of Phospho-ERK1/2, the membrane was stripped with stripping buffer (0.05 M Tris pH 6.8, 2% SDS, 0.8% beta-mercaptoethanol) for 30 min at 50 °C before detecting total ERK1 + ERK2.

### 4.9 Immunofluorescence analyses (IFA)

10,000 cells were seeded onto a 13 mm glass coverslip in 24-well glass bottom plate coated with 10 ng/ml fibronectin. The next day, medium was replaced to starved medium and cultured for 1 h, then the cells were stimulated for indicated times and fixed with 4% paraformaldehyde. After washing 3 times by PBS at room temperature, cells were permeabilized with 0.5% Triton X-100 in PBS for 10 min. Blocking of nonspecific epitopes was performed in blocking buffer (10% FBS in PBS) for 15 min at room temperature. The primary antibodies were applied at 1:100 dilutions in blocking buffer at 4 °C overnight. The fluorophore-conjugated secondary antibody (Donkey anti Rabbit IgG (H + L), Alexa Fluor 488 Thermo Fisher Scientific A-21206) and Hoechst 33342 were applied at 1:1000 dilutions in blocking buffer for 1–2 h at room temperature in the dark. Images were acquired by Zeiss LSM 880 confocal microscope, and the images were processed with the FIJI software.

### 4.10 Animal experiment: intradermal ear injection and imaging

Mouse-ear injections of cells were carried out in 10-week-old male NOD/SCID mice with interleukin-2 receptor gamma chain null mutation (Il2rg −/−). SCC cells expressing the ERK-SKARS-mCherry or ERK-SKARS-GFP and MEK2_ND_-SKARS-mCherry were cultured in 10 cm dishes for 50–60 % confluence. After trypsinization and centrifugation, SCC13 cells were resuspended in 3 μl of sterile Hank's Balanced Salt Solution, and injected intradermally in mouse ears through a 33-gauge microsyringe (Hamilton). Mice were sacrificed 3 weeks later for live tissue imaging analysis. Fresh tumors were sectioned into <1 mm slices and further analyzed through an inverted confocal microscope (Zeiss LSM880).

## Declarations

### Author contribution statement

M. Ma: Conceived and designed the experiments; Performed the experiments; Analyzed and interpreted the data; Wrote the paper.

S. Pelet: Conceived and designed the experiments; Analyzed and interpreted the data; Wrote the paper.

G. Dotto: Conceived and designed the experiments.

P. Bordignon: Performed the experiments.

### Funding statement

This work was supported by 10.13039/100000001Swiss National Science Foundation (SNSF) and the 10.13039/501100006390University of Lausanne. M. Ma was supported by the Faculty of Biology and Medicine (FBM) interdisciplinary grant from the 10.13039/501100006390University of Lausanne.

### Data availability statement

Data will be made available on request.

### Declaration of interests statement

The authors declare no conflict of interest.

### Additional information

No additional information is available for this paper.

## Appendix A Supplementary data

Supplementary_Material_SP201026.pdf Supplementary_Material_SP201026.pdf
