# Supplementary material for: Visualizing cellular heterogeneity by quantifying the dynamics of MAPK activity in live mammalian cells with synthetic fluorescent biosensors
Source: Heliyon. 2020 Dec 7;6(12):e05574. doi: 10.1016/j.heliyon.2020.e05574 (PMC7723811; doi:10.1016/j.heliyon.2020.e05574)
Supplement: Supplementary_Material_SP201026.pdf [file mmc1.pdf]

Supplementary Material to

Visualizing cellular heterogeneity by quantifying the dynamics of MAPK activity in live mammalian cells with synthetic fluorescent biosensors

Min Ma, Pino Bordignon, Gian-Paolo Dotto and Serge Pelet

Supplemental information

Supplementary Figures 1-8

Supplementary Movie 1

Supplementary Tables 1-2

Supplementary Figure Legends

Supplementary Figure 1. ERK activity measured by Western blot and immunofluorescence

a. and b. Representative immunofluorescence microscopy images of HeLa cells exposed to EGF (50 ng/ml) stimulation for the indicated period of time and probed for phosphorylated ERK (a) or total ERK levels (b). c. Time course analysis of total and phosphorylated ERK by Western Blot upon stimulation of Hela cells with 50 ng/ml EGF.

Supplementary Figure 2. Characterization of the NLS and docking site specificity for ERK-SKARS function.

a. Amino acid sequence of NLS-2A and NLS-2E mutation. b. Cellular translocation of ERK-SKARS requires phosphorylatable NLS. After quantification of the time-lapse movies, Cyto/Nucl ratio of HeLa cells is plotted as function of time. HeLa cells expressing ERK-SKARS (green), ERK-SKARS^{4A} (blue) and ERK-SKARS^{4E} (red) were stimulated with EGF 50 ng/ml. The solid lines represent the median of the cell population and the shaded area the 25 and 75 percentiles of the population. More than 100 single cells were used to plot the graph. c. Amino acid sequence of the non-functional docking site of MEK2 for ERK. d. Microscopy images of HeLa cells co-expressing ERK-SKARS^{DS} (green) and ERK-SKARSND (red) exposed to EGF stimulation (50 ng/ml). HeLa cell nuclei were stained with Hoescht (cyan). e. Quantification of the time-lapse movie shown in d. The Cyto/Nucl ratio of the functional (green) and non-functional (red) sensors averaged over at least 120 single cells f. Few single cell measurements of the normalized Cyto/Nucl ratio. Each color corresponds to one single cell. For this cell, the solid line represents the ERK-SKARS^{DS} measured in the green channel and the dashed line represent the ERK-SKARSND in the red channel.

Supplementary Figure 3. ERK-SKARS specificity validated using MAPK inhibitors.

a. to c. HeLa cells expressing the ERK-SKARS were pre-incubated with DMSO (red), or ERK inhibitor (PD032591 100 nM, a, blue), p38 inhibitor (10 uM p38 inhibitor, b, green) and JNK inhibitor (10 uM JNK inhibitor VIII, c, yellow) for 30 min before imaging. Cells were subsequently stimulated with EGF 50 ng/ml.

Supplementary Figure 4. Images of JNK-SKARS and p38-SKARS sensors.

a. Specific sequence used for developing JNK. The JNK-SKARS is composed of the c-Jun docking site (c-Jun, amino acids 29-57), double NLS and the mCherry protein. b. Microscopy images of HeLa cells expressing the JNK-SKARS and stimulated with Anisomycin (50 ng/ml). c. The p38-SKARS is composed of the Mef2C docking site (Mef2C, amino acids 251-278), double NLS and the mCherry protein. c. Microscopy images of HeLa cells expressing the p38-SKARS and stimulated with Anisomycin (50 ng/ml).

Supplementary Figure 5. Comparison of the SKARS and KTR reporters.

a. Comparison of the relative fluorescence intensities of the ERK-SKARS-mCherry (red) and the ERK-KTR-mClover (green). Cells expressing both sensors within the range of fluorescence intensities indicated by the arrows were kept for analysis. b. Nuclear enrichment (Nucl/Cyto ratio) of the ERK-SKARS (red) and the ERK-KTR (green) before (solid line) and at the end of the time lapse (dashed line). c. Median of the Cyto/Nucl ratios from ERK-SKARS (red) and ERK-KTR (green) upon stimulation with 10 ng/ml EGF (dashed line, three biological replicates) and 0 ng/ml EGF (solid line). At least 160 single cell traces were measured for each replicate. The single cell traces were normalized to the basal level (the mean of the first 3 three time points). d. Violin plot of the normalized initial response of the ERK-SKARS (red) and ERK-KTR (green). Each dot represents one single cell measurement. The solid line is the median of the population. The asterisks denote a significant difference between the SKARS and KTR measurements based on a Wilcoxon rank sum test ($P < 0.005$)

Supplementary Figure 6. Comparison of JNK-SKARS and JNK-KTR.

a. Microscopy images of HeLa cells carrying JNK-SKARS-mCherry (red) and JNK-KTR-mClover (green) exposed to Anisomycin (50 ng/ml) stimulation and imaged at indicated time points. b. After quantification of the time-lapse movies, traces of Cyto/Nucl ratio from JNK-SKARS (red) and JNK-KTR (green) normalized by the average of the first 3 time points are plotted as function of time. c. Histograms of the cell nuclear fluorescence intensity in the GFP (JNK-KTR) and RFP (JNK-SKARS) channels. 134 cells expressing both sensors within the range of fluorescence intensities indicated by the arrows were kept for analysis. d. Few single cell measurements of the normalized Cyto/Nucl ratio following Anisomycin (50 ng/ml) stimulation. Each color corresponds to one single cell. For this cell, the solid line represents the ERK-SKARS measured in the red channel and the dashed line represent the ERK-KTR in the green channel.

Supplementary Figure 7. Single cell responses to high doses of EGF for two of the five clusters

a. and b. Microscopy images of HeLa cells expressing the ERK-SKARS from two different sub-populations (C and E). The left panel displays the Cyto/Nucl measurements from four single cells from that sub-population (dashed lines). The thin solid line corresponds to the single cell identified in the microscopy images on the left with an asterisk. The thick solid line represents the mean response from the sub-population.

Supplementary Figure 8. Analysis of pulses in single cell responding to low doses of EGF.

a. Median Cyto/Nucl ratio of a clonal population of Hela cells expressing the ERK-SKARS responding to low concentrations of EGF (Nc > 300) for more than 6 hours. b. Median (solid line) and 25- to 75-percentiles (area) of the Cyto/Nucl ratio of ERK-SKARS in more than 400 single cells from a clonal HeLa cell population stimulated with low doses of EGF and monitored for more than 3 hours. c. Violin plot of the initial response of the ERK-SKARS at various concentrations. Each dot represents one single cell measurement. The solid line is the median of the population. The asterisks denote a significant difference from the 0.0 ng/ml EGF control sample based on a Wilcoxon rank sum test ($P < 0.005$). d. Histogram of the sum of the amplitudes of all the pulses in the traces displaying more than 2 pulses. The cell count in the histogram was normalized to the total number of cells measured in the dataset. e. Histogram of the number of peaks detected at the different concentrations of EGF.

Supplementary Movie 1. Pulses of ERK activity

Time-lapse movie of HeLa cells expressing the ERK-SKARS displaying pulses in ERK activation upon 0.1 ng/ml EGF stimulation.

Supplementary Figure 1

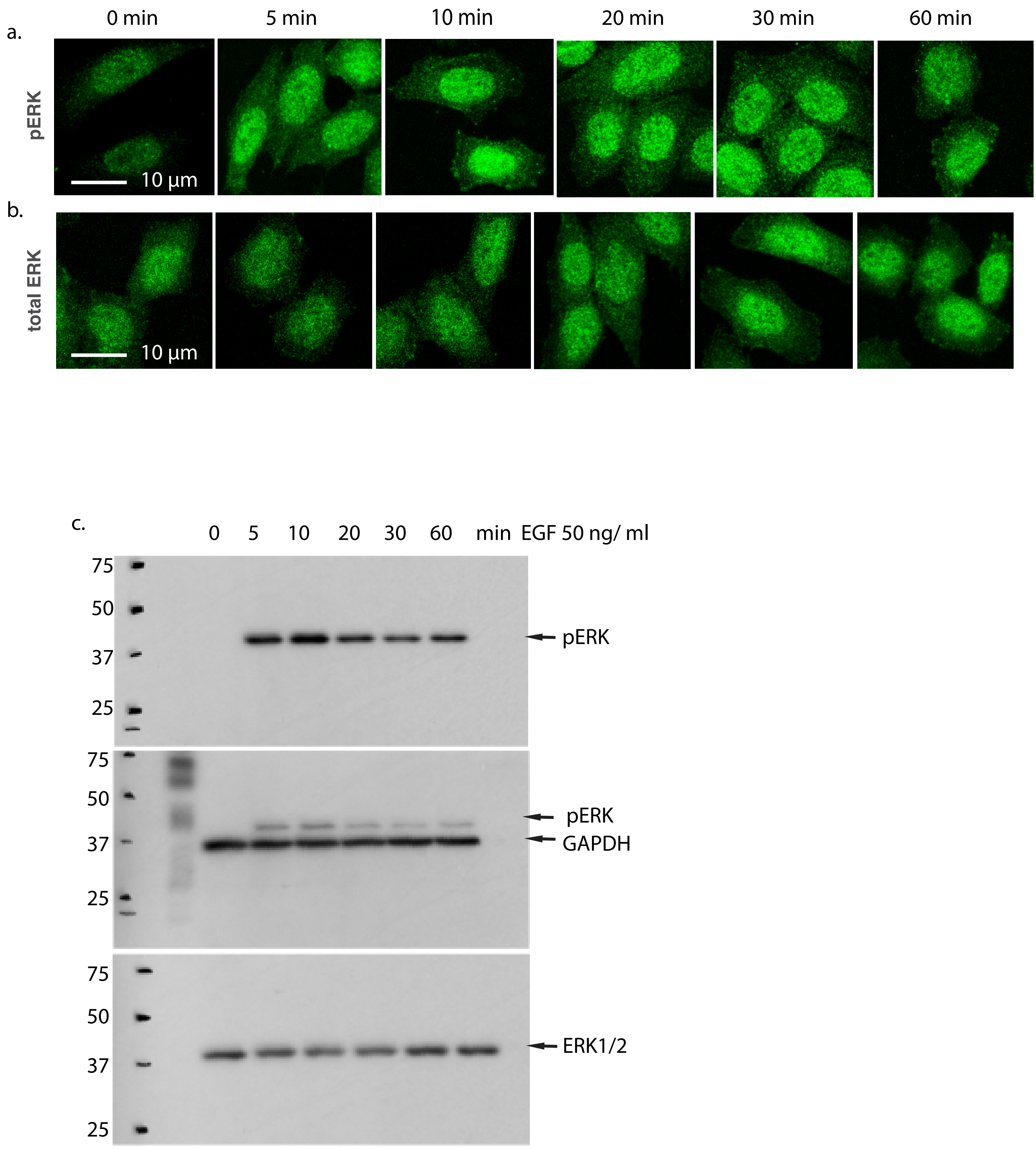

Supplementary Figure 2

a.

NLS-2A: ELGAPLKKLKIAPDT

NLS-2E: ELGEPLKKLKIEPDT

b.

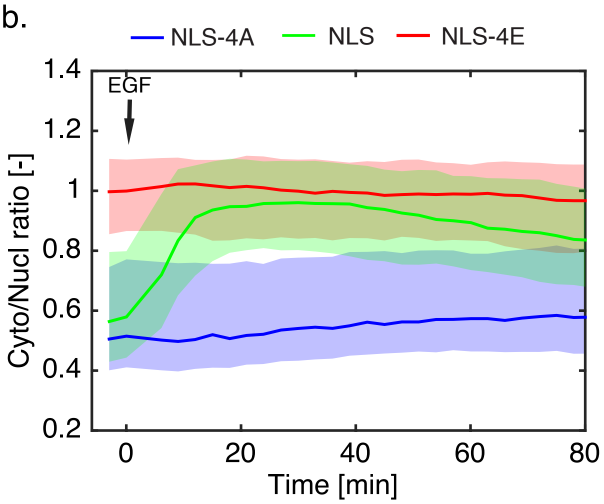

c.

$MEK2_{ND}$:

MLAAAEPVLPAATANPTIAEGPSPTSEGASEANLVDLQKK

d.

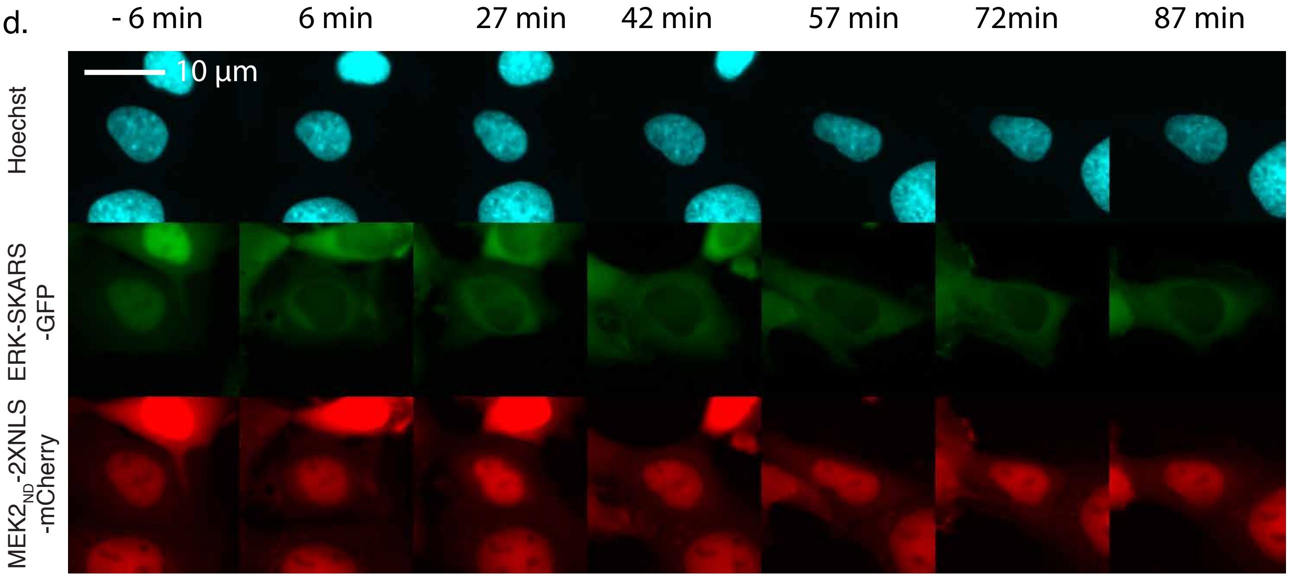

e.

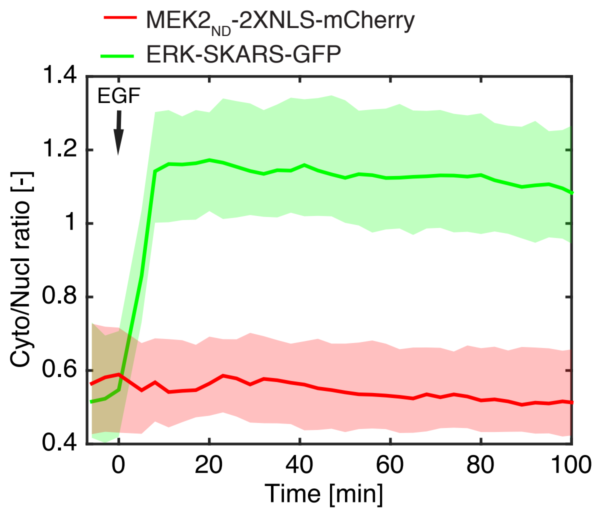

f.

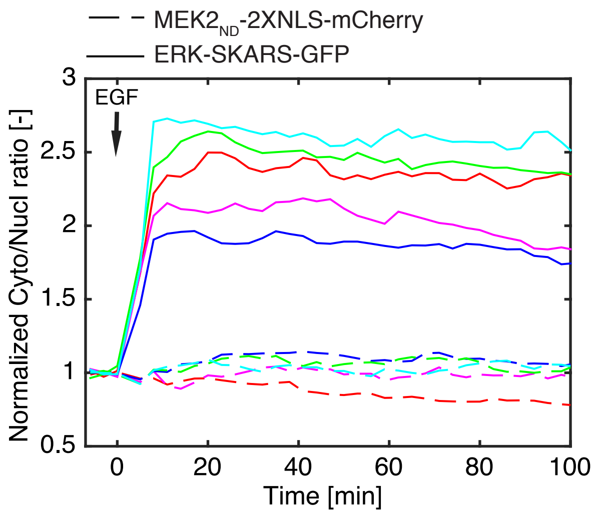

Supplementary Figure 3

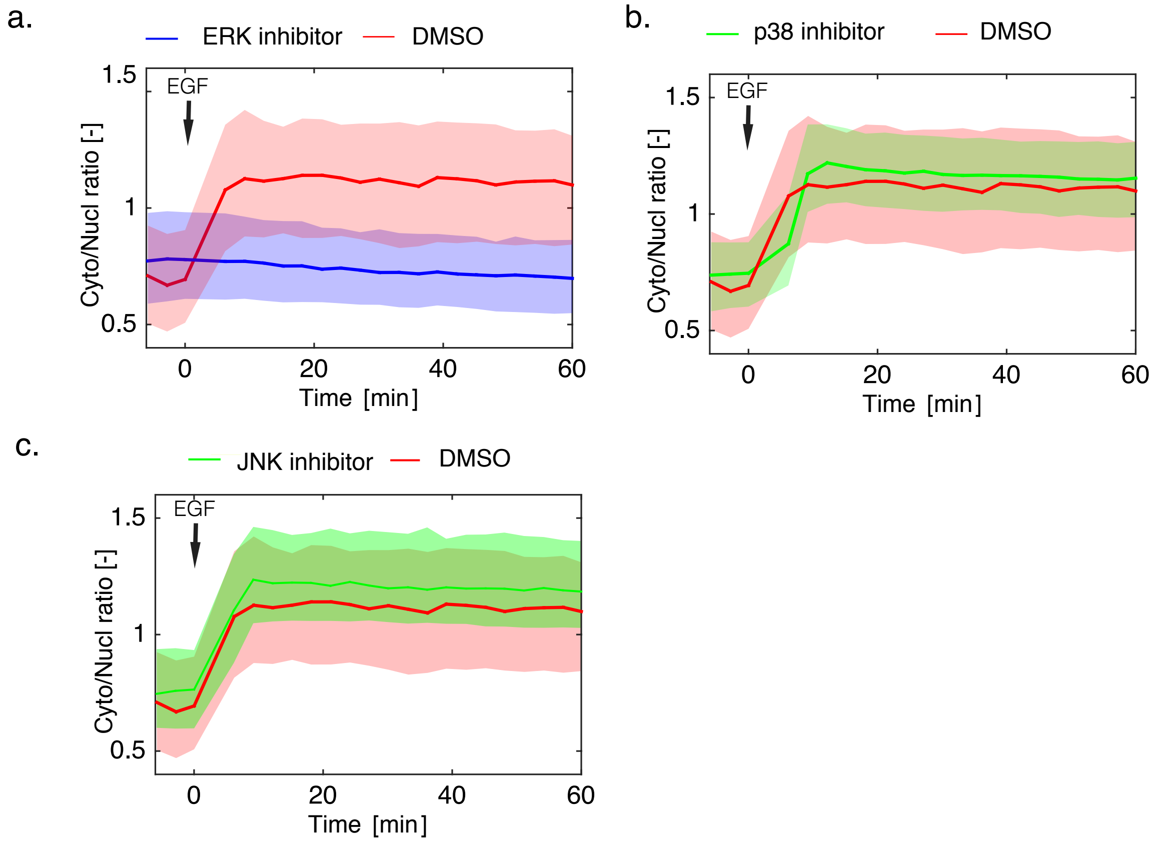

Supplementary Figure 4

a. JNK-SKARS

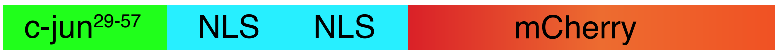

c-Jun DS:
MSNPKILKQSMTLNLADPVGSLKPHLRA

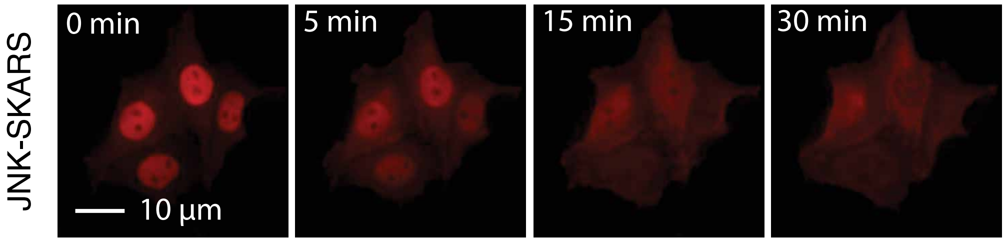

b. p38-SKARS

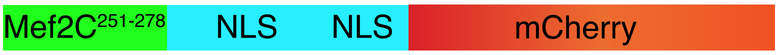

Mef2C DS:
MRKPDLRVLIPPGSKNTMPSVNQRINNS

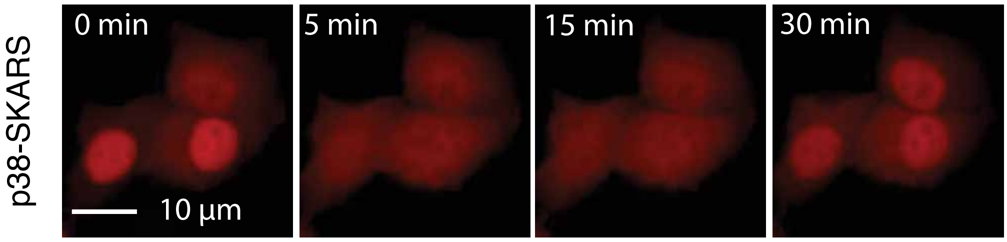

Supplementary Figure 5

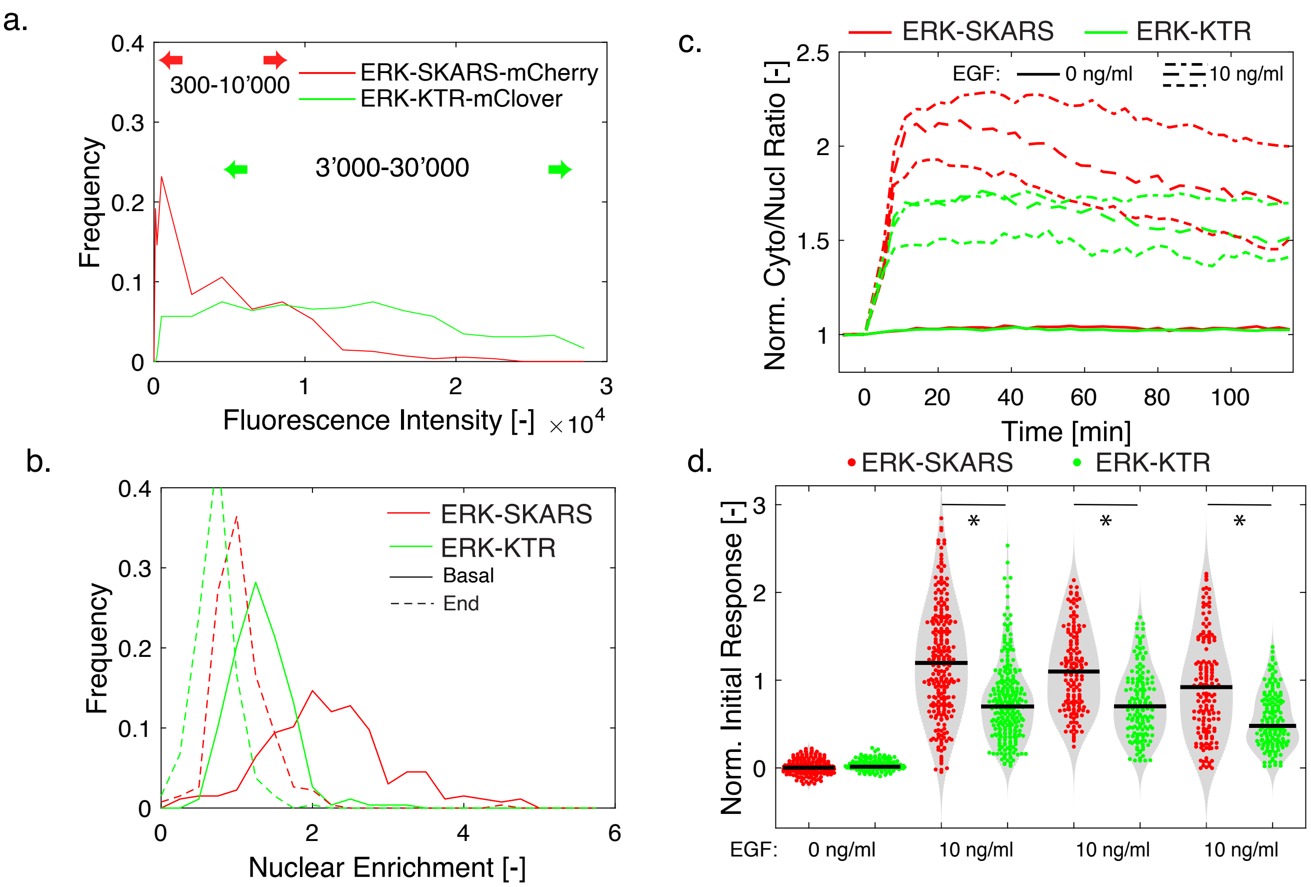

Supplementary Figure 6

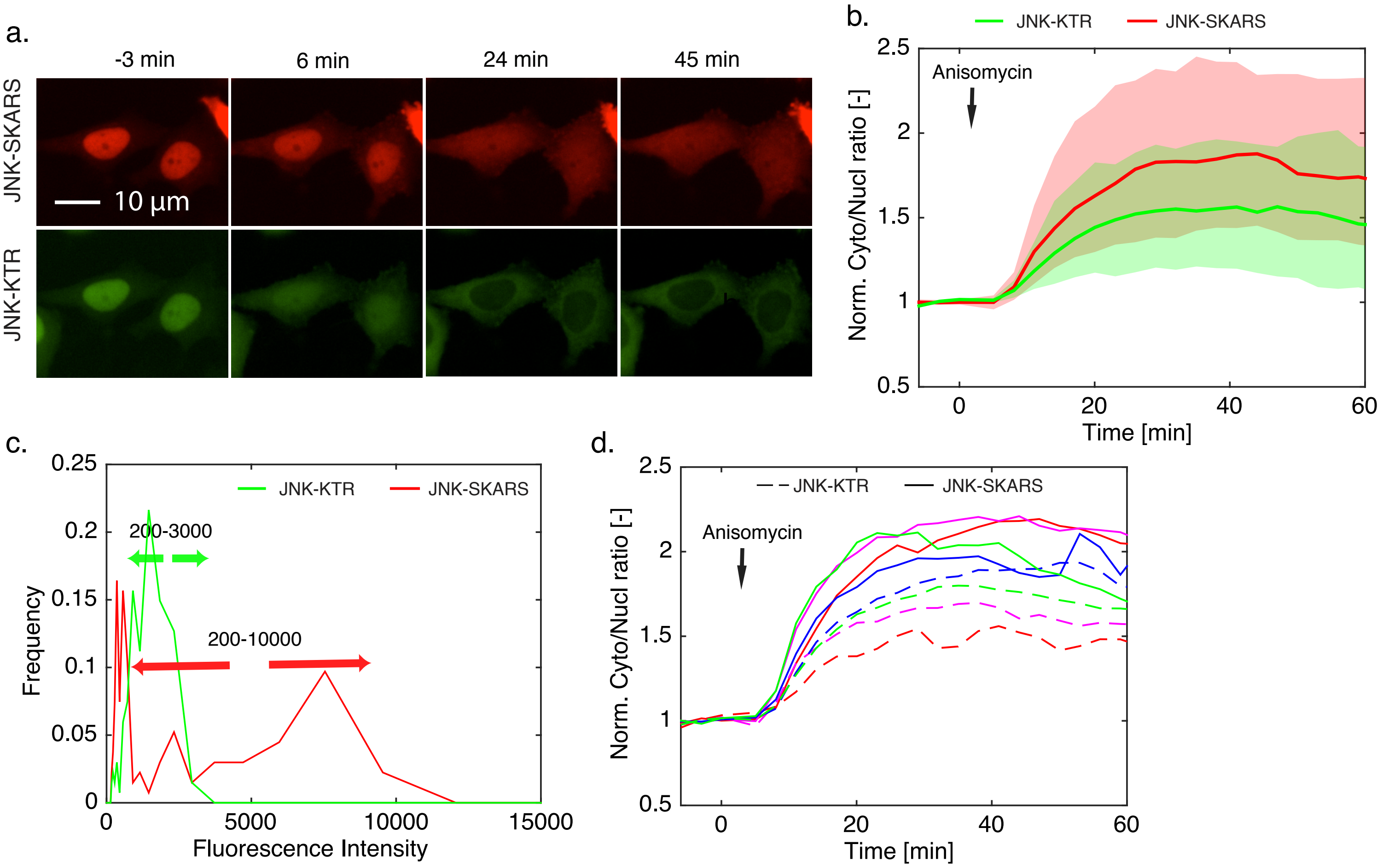

Supplementary Figure 7

a.

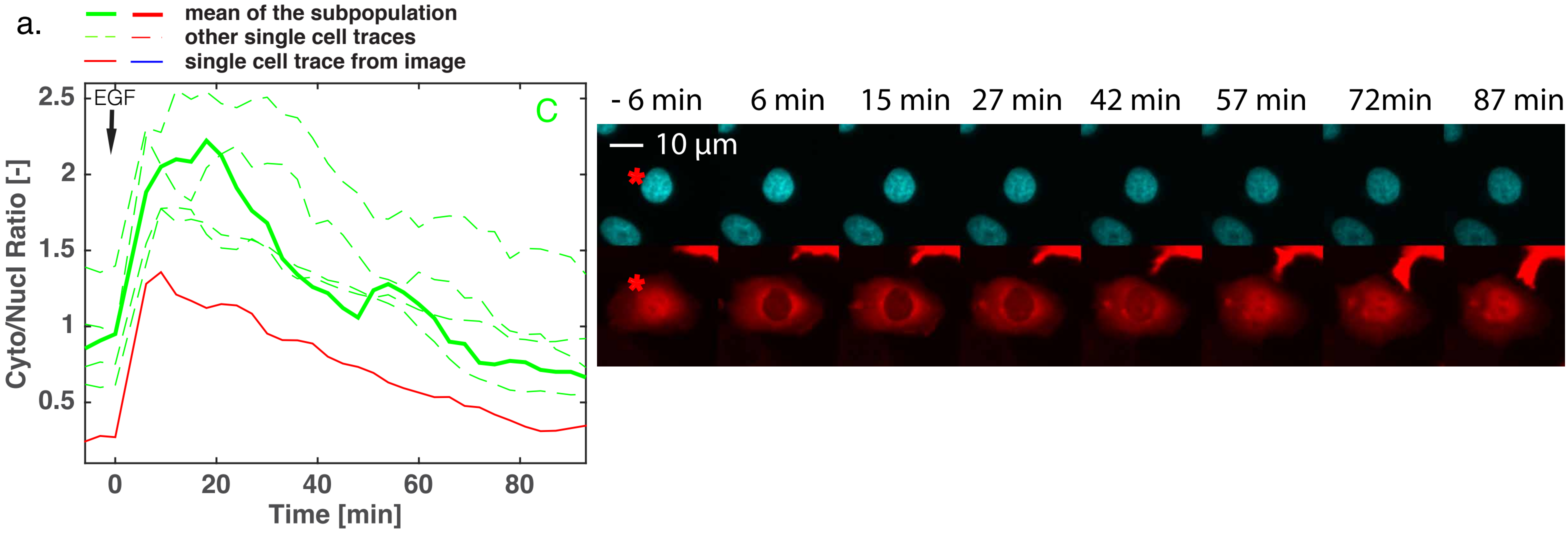

b.

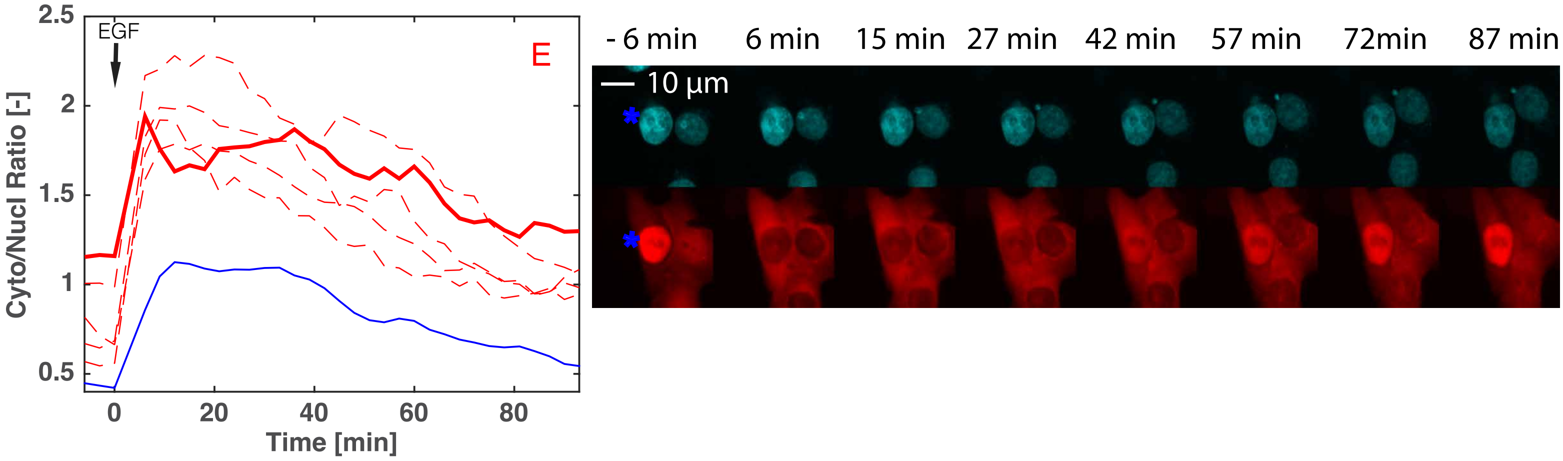

Supplementary Figure 8

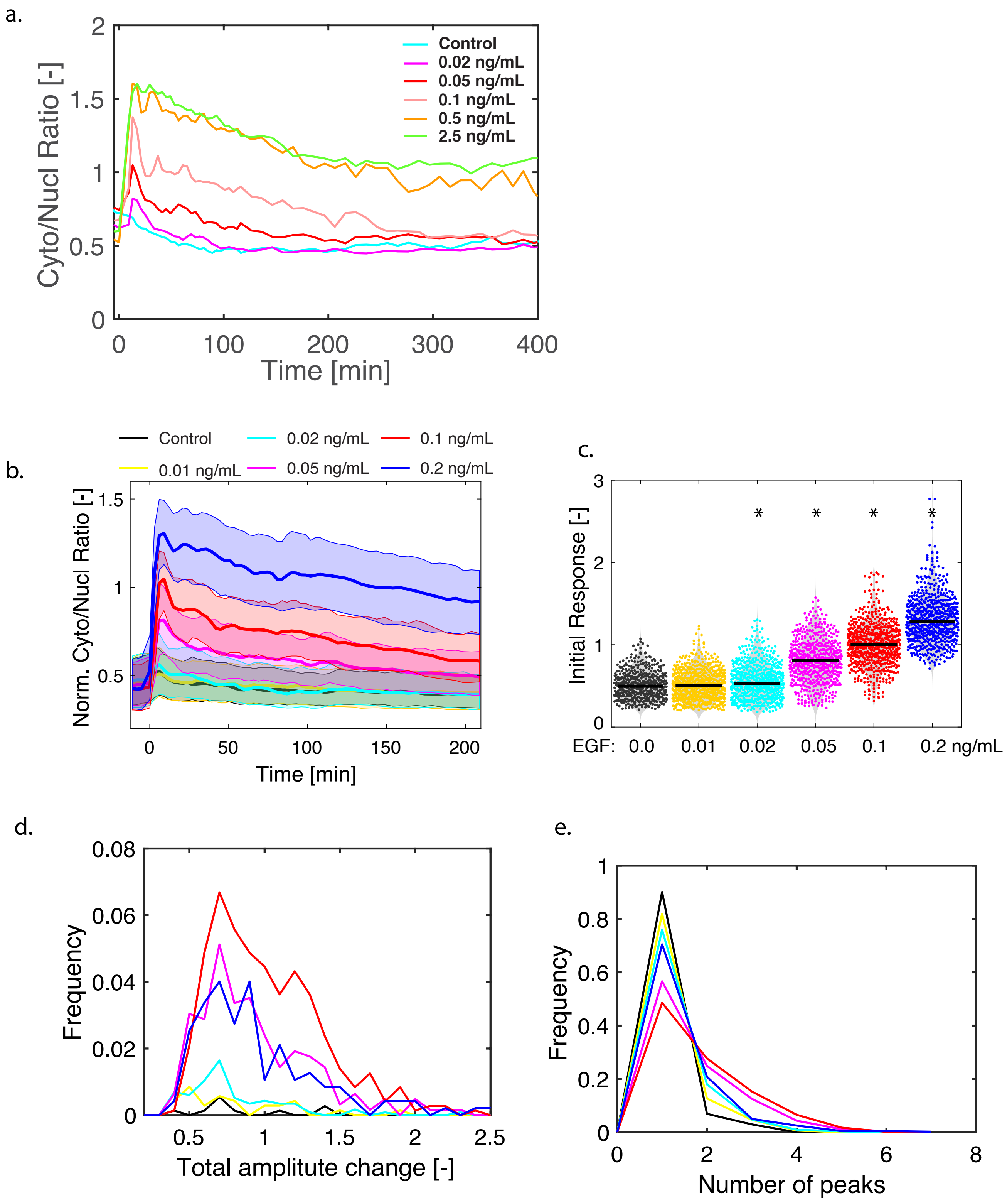

Supplementary Table 1. Comparison between the SKARS and KTR responses.

Experiments performed on three biological replicates treated either with 0 or 10 ng/ml of EGF. The median and the 25- and 75- percentiles is provided for each measurement.

	Replicate # 1 0ng/ml EGF		Replicate # 2 0ng/ml EGF		Replicate # 3 0ng/ml EGF		Replicate # 1 10ng/ml EGF		Replicate # 2 10ng/ml EGF		Replicate # 3 10ng/ml EGF	
**Initial Response SKARS**	0.014	-0.004	0.008	-0.024	0.003	-0.037	0.499	0.344	0.511	0.395	0.450	0.264
		0.036		0.050		0.038		0.651		0.655		0.622
**Initiale Response KTR**	0.020	-0.008	0.026	-0.006	0.012	-0.003	0.496	0.325	0.478	0.310	0.374	0.248
		0.046		0.077		0.051		0.659		0.632		0.559
**Normalized Initial Response SKARS**	0.029	-0.005	0.017	-0.042	0.004	-0.056	1.203	0.708	1.103	0.672	0.923	0.429
		0.083		0.091		0.082		1.688		1.548		1.462
**Normalized Initial Response KTR**	0.029	-0.009	0.030	-0.007	0.014	-0.005	0.701	0.396	0.704	0.385	0.482	0.275
		0.065		0.099		0.069		0.991		1.014		0.814
**Basal Level SKARS**	0.462	0.357	0.549	0.436	0.521	0.427	0.431	0.349	0.492	0.364	0.521	0.398
		0.678		0.737		0.706		0.546		0.621		0.671
**Basal Level KTR**	0.735	0.601	0.824	0.711	0.824	0.686	0.711	0.596	0.705	0.614	0.770	0.638
		0.876		0.962		0.961		0.867		0.802		0.884
**Level 5min SKARS**	0.476	0.380	0.559	0.446	0.526	0.416	0.957	0.787	1.028	0.863	1.027	0.852
		0.704		0.717		0.717		1.137		1.233		1.186
**Level 5min KTR**	0.760	0.643	0.874	0.758	0.866	0.712	1.214	1.027	1.170	1.058	1.147	1.019
		0.890		0.972		0.972		1.436		1.349		1.314
**Level 100min SKARS**	0.509	0.386	0.543	0.438	0.515	0.409	0.895	0.728	0.831	0.706	0.814	0.643
		0.701		0.676		0.661		1.043		1.007		0.969
**Level 100min KTR**	0.770	0.636	0.833	0.715	0.816	0.672	1.185	1.013	1.076	0.946	1.091	0.941
		0.903		0.950		0.915		1.447		1.273		1.267
**Number of cells**	201		134		148		234		148		157	

Supplementary Table 2. Resources used in this study

REAGENT or RESOURCE	SOURCE	IDENTIFIER
**Chemicals, antibodies, recombinant peptides**		
AEG 3482	Sigma-aldrich	SML0264-5MG
p38 MAP kinase inhibitor IV	Sigma-aldrich	SML0543-5MG
FR180204	Sigma-aldrich	SML0320-5MG
Fibronictin	ThermoFisher Scientific	Gibco 33010018
JetPEI	Polyplus transfection	101-10N
Anisomycin	Sigma-aldrich	A9789-5MG
Recombinant human FGF-basic (154a.a)	ThermoFisher Scientific	Gibco PHG0024
EGF recombinant human protein	ThermoFisher Scientific	PHG0311
Paraformaldehyde	Sigma-aldrich	158172-500G
Hoechst 33342	Molecular Probes	H3579
Fibronectin	ThermoFisher Scientific	33010-018
Polybrene	Sigma-aldrich	AL-118
Phospho-p44/p42 MAPK (Erk1/2) (Thr202/tyr204) (D13.14.4E) XP Rabbit mAB	Cell Signaling Technology	4370
Rabbit anti-ERK1 Antibody (C-16)	Santa Cruz	sc-93
Rabbit anti-ERK2 Antibody (C-14)	Santa Cruz	sc-154
GAPDH FL-335	Santa Cruz	sc-25778
Donkey anti Rabbit IgG (H +L), Alexa Fluor 488	ThermoFisher Scientific	A-21206
Rabbit IgG, HRP-linked whole Ab	Amersham	NA 934
Dulbecco's modified Eagle's medium	ThermoFisher Scientific	31966021
Gibco FluoroBrite DMEM medium	ThermoFisher Scientific	A1896701
fetal bovine serum	ThermoFisher Scientific	10270106
Antibiotics	ThermoFisher Scientific	15240062
Puromycin	ThermoFisher Scientific	A1113803
**Experimental Models: Cell Lines**		
293T	ATCC	CRL-11268
HeLa	ATCC	CCL-2
SCC13	RRID	CVCL-4029
MDA-MB-231	ATCC	HTB-26
MCF7	ATCC	HTB-22
Hela-ERK-SKARS	This paper	N/A
Hela-JNK-SKARS	This paper	N/A
Hela-p38-SKARS	This paper	N/A
SCC13-ERK-SKARS	This paper	N/A
MDA-MB-231-ERK-SKARS	This paper	N/A
MCF7-ERK-SKARS	This paper	N/A
Hela-ERK-KTR/SKARS	This paper	N/A
HeLa-JNK-KTR/SKARS	This paper	N/A
SCC13-ERK-SKARS ND/DS	This paper	N/A
Hela-ERK-SKARS SC1	This paper	N/A
**Experimental models: Primary cells**		
HFF	CHUV	N/A
**Experimental models: strain**		
mouse		
**Recombinant DNA (plasmid)**		
pLV-CMV-MEK2-2NLS-mCherry	This paper	pMM16
pLV-CMV-cJun-2NLS-mCherry	This paper	pMM65
pLV-CMV-cMef2C-2NLS-mCherry	This paper	pMM66
pLV-CMV-MEK2ND-2NLS-mCherry	This paper	pMM26
pLV-CMV-MEK2-2NLS(4A)-mCherry	This paper	pMM30
pLV-CMV-MEK2-2NLS(4E)-mCherry	This paper	pMM31
pLV-CMV-MEK2-2NLS-mClover	This paper	pMM55
pLentiCMV Puro DEST ERKKTRClover	Regot et al. 2014	Addgene 59150
pLentiCMV Puro DEST JNKKTRClover	Regot et al. 2014	Addgene 59151
**Software and Algorithms**		
Micro-manager	Edelstein et al, 2010	ver 1.4
FIJI Image J	http//fiji.sc/	Ver 2.0
YeastQuant platform	Pelet et al., 2012	Ver X.2
Matlab software	Mathworks	R2017b
